# Subversion of Cell-Autonomous Immunity and Cell Migration by *Legionella pneumophila* Effectors

**DOI:** 10.3389/fimmu.2015.00447

**Published:** 2015-09-14

**Authors:** Sylvia Simon, Hubert Hilbi

**Affiliations:** ^1^Institute of Medical Microbiology, University of Zürich, Zürich, Switzerland; ^2^Max von Pettenkofer Institute, Ludwig-Maximilians University, Munich, Germany

**Keywords:** bacterial pathogenesis, *Dictyostelium*, inflammasome, *Legionella*, macrophage, pathogen vacuole, phosphoinositide, small GTPase

## Abstract

Bacteria trigger host defense and inflammatory processes, such as cytokine production, pyroptosis, and the chemotactic migration of immune cells toward the source of infection. However, a number of pathogens interfere with these immune functions by producing specific so-called “effector” proteins, which are delivered to host cells via dedicated secretion systems. Air-borne *Legionella pneumophila* bacteria trigger an acute and potential fatal inflammation in the lung termed Legionnaires’ disease. The opportunistic pathogen *L. pneumophila* is a natural parasite of free-living amoebae, but also replicates in alveolar macrophages and accidentally infects humans. The bacteria employ the intracellular multiplication/defective for organelle trafficking (Icm/Dot) type IV secretion system and as many as 300 different effector proteins to govern host–cell interactions and establish in phagocytes an intracellular replication niche, the *Legionella*-containing vacuole. Some Icm/Dot-translocated effector proteins target cell-autonomous immunity or cell migration, i.e., they interfere with (i) endocytic, secretory, or retrograde vesicle trafficking pathways, (ii) organelle or cell motility, (iii) the inflammasome and programed cell death, or (iv) the transcription factor NF-κB. Here, we review recent mechanistic insights into the subversion of cellular immune functions by *L. pneumophila*.

## Introduction

Phagocytic cells of the innate immune system, such as macrophages, neutrophils, and dendritic cells (DC), produce pathogen recognition receptors (PRRs) comprising families of membrane-bound or cytosolic receptors (1–4). The membrane-bound receptors are toll-like receptors (TLRs) and C-type lectin receptors (CLRs), such as the mannose receptors. TLRs trigger cytokine production by signaling through the adaptor molecules MyD88 or TRIF and activate the transcription factor NF-κB or MAP kinase pathways. Cytosolic receptors are Nod-like receptors (NLRs) involved in inflammatory responses and cell death pathways through the activation of multiprotein complexes termed inflammasomes (5), as well as retinoic acid inducible gene-I (RIG-I)-like receptors (RLRs). Collectively, these receptors recognize pathogen-associated molecular patterns (PAMPs) and danger-associated molecular patterns (DAMPs), thus alerting the infected host cell, if material arising from cell infection or cell damage is present. Subsequent cytokine production promotes the clearance of invading microorganisms. The conserved process of cell-autonomous immunity includes not only inflammasome activation and interleukin (IL)-1β production, as well as autophagy, but also NF-κB- and type I interferon (IFN)-dependent cytokine production (6, 7).

Pathogenic bacteria target eukaryotic cells either as adversaries in the case of potentially bactericidal cells of the metazoan immune system and/or as a rich source of nutrients (8, 9). Accordingly, pathogens developed means to counteract the arsenal of the humoral and cellular components of the innate and acquired immune system and to survive and replicate within eukaryotic cells, including phagocytes (10). To this end, a number of pathogens produce specific so-called “effector” proteins, which are delivered via dedicated secretion systems into host cells, where they interfere with immune functions and cell migration (11).

*Legionella pneumophila* employs the intracellular multiplication/defective for organelle trafficking (Icm/Dot) type IV secretion system (T4SS) and as many as 300 different effector proteins to govern host–cell interactions. The role and molecular mode of action of effectors involved in pathogen vacuole formation has recently been reviewed in detail (12). Here, we will review mechanistic insights into the subversion of cell-autonomous immunity and cell migration by *L. pneumophila*.

## Pathogenesis of *Legionella pneumophila*

*Legionella pneumophila* is an opportunistic pathogen and causes a severe pneumonia termed Legionnaires’ disease. The Gram-negative genus *Legionella* comprises more than 55 species with several serogroups; yet, at least 85% of human infections are caused by *L. pneumophila* (13). Evolutionary adaptation allows *L. pneumophila* to persist in a variety of extra- and intracellular niches. The aerobic bacterium can not only replicate in biofilms but also resists degradation by free-living protozoa and replicates within, e.g., *Acanthamoeba*, *Hartmannella*, and *Tetrahymena* species, as well as in *Dictyostelium discoideum*, although the latter amoeba is likely not a natural host (14, 15). *L. pneumophila* is ubiquitously found in natural and technical water systems, including cooling towers, whirlpools, and showers.

Upon inhalation of contaminated aerosols, *L. pneumophila* resists degradation and replicates within alveolar macrophages, which is a precondition for the onset of disease (16). The acquisition of the pathogen from environmental sources is the only infection route; transmission between humans has never been observed. Since *L. pneumophila* probably mainly evolved as a parasite of free-living protozoa, the human host represents a dead-end for this “accidental” pathogen. Thus, *L. pneumophila* likely has not been exposed to a rigorous evolutionary selection to avoid recognition by mammalian PRRs, and accordingly, the bacteria trigger the activation of all PRR families (17).

Most humans and mice are able to clear a *Legionella* infection, and therefore, the development of a suitable small animal model was crucial. Initial studies using guinea pigs exposed to *L. pneumophila*-containing aerosols revealed a high susceptibility of these animals, which developed an illness reminiscent of typical human Legionnaires’ disease (18). While most inbred mouse strains are resistant to *L. pneumophila* infection and disease progression, the A/J mouse strain was found to be susceptible and to present with acute pneumonia that resembled human disease (19). These results correspond to *in vitro* infections of peritoneal mouse ­macrophages, which indicated that cells from A/J mice were much more permissive for intracellular replication of *L. pneumophila* than macrophages from other mouse strains, such as C57BL/6 and BALB/c (20, 21). Macrophages from C57BL/6 and BALB/c mice restrict *L. pneumophila* by the activation of a programed cell death pathway as an ultimate line of defense against the intracellular pathogen (see below). Accordingly, mice lacking components of this pathway fail to restrict *L. pneumophila* and faithfully mimic *Legionella* pathology (17, 22).

In A/J mice, *L. pneumophila* elicits an acute inflammatory reaction, including production of the cytokines tumor necrosis factor (TNF)-α, IFN-γ, IL-12, and IL-18, which restrict pathogen replication (17, 22). IFN-γ is particularly important to inhibit bacterial growth in monocytes and alveolar macrophages, thus contributing to limiting the infection by *L. pneumophila* (23–25). These inflammatory cytokines recruit and activate polymorphonuclear neutrophil granulocytes (PMNs) (26–28). PMNs are central innate effector cells that not only resolve *L. pneumophila* infection but – in concert with IFN-γ-producing natural killer (NK) cells – also secrete cytokines, such as IL-18 (29, 30). In a feedback loop, IFN-γ triggers IL-12 production by DC, which activate NK cells. Therefore, DC are also essential to control *L. pneumophila* infection (31). Interestingly, DC restrict the intracellular growth of *L. pneumophila*, despite that the pathogen resides in an apparently non-bactericidal compartment derived from the endoplasmic reticulum (ER) (32).

## Formation of the Intracellular Replication Niche

**Figure 1:**
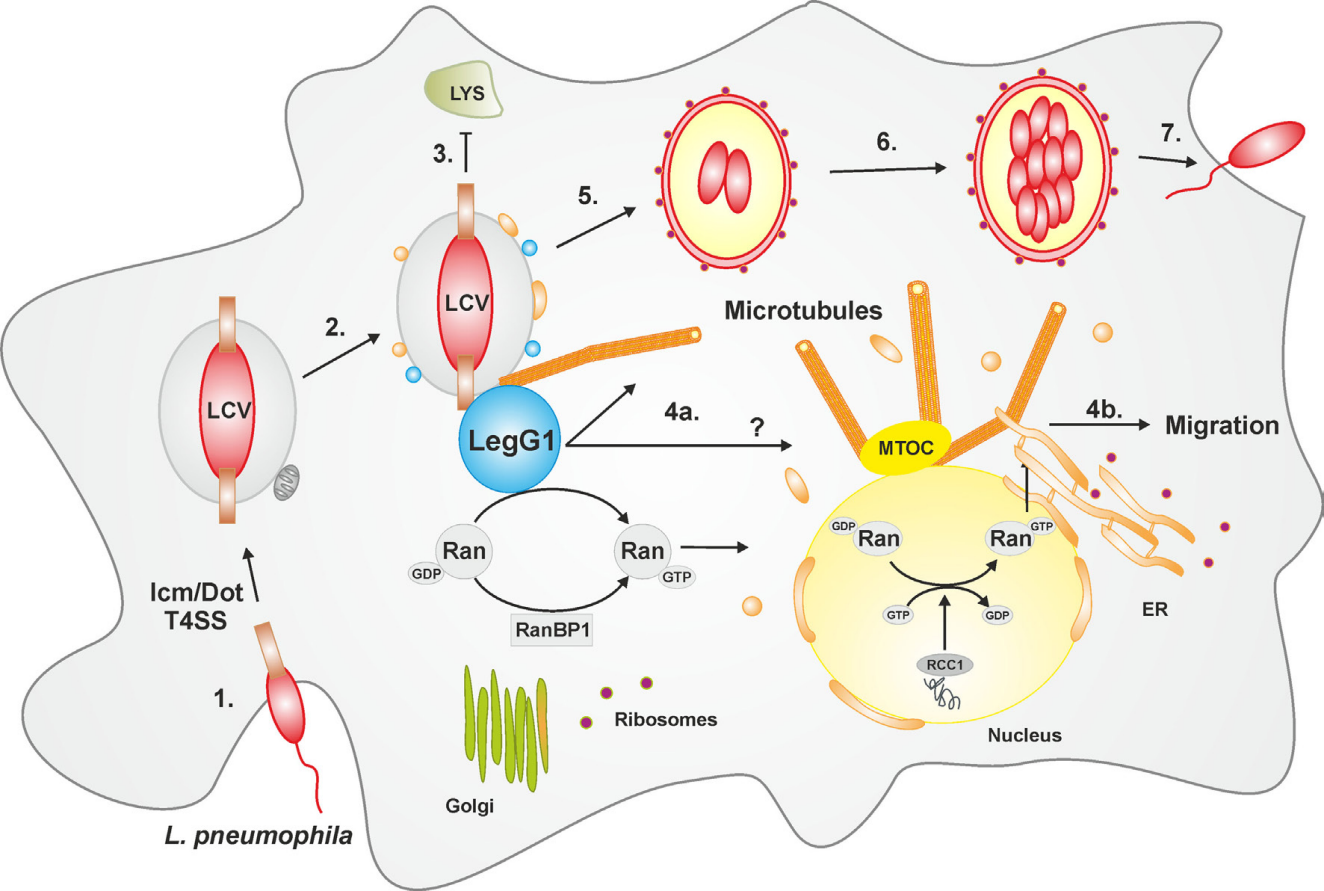
**LCV formation and LegG1-dependent modulation of cell migration**. The intracellular phases of *L. pneumophila* can be divided in seven main steps: (1) Adhesion and entry into the host cell via macropinocytosis; (2) Formation of the LCV in an Icm/Dot T4SS-dependent manner and recruitment of vesicles from the ER as well as mitochondria; (3) Evasion from the lysosomal trafficking; (4a) The bacterial effector protein LegG1 activates the GTPase Ran, stabilizes microtubules in the vicinity of the LCV or possibly at a distance (?), and (4b) promotes cell migration. In the nucleus, Ran activation is triggered by the eukaryotic GEF RCC1. (5) The LCV communicates with host vesicle trafficking pathways, and acquires and eventually fuses with the ER; (6) The pathogen compartment turns in a rough-ER-like vacuole, wherein the bacteria replicate; (7) *L. pneumophila* is released and re-infects new host cells or is transmitted to other environmental niches. Abbreviations: ER, endoplasmic reticulum; LCV, *Legionella*-containing vacuole; MTOC, microtubule organizing center; Ran, Ras-related nuclear protein; RCC1, regulator of chromosome condensation 1; T4SS, type IV secretion system.

In permissive macrophages as well as in protozoa, *L. pneumophila* employs a complex and apparently evolutionarily conserved mechanism to establish its replication-permissive membrane-bound niche, the *Legionella*-containing vacuole (LCV) (Figure 1). LCV formation is governed by the Icm/Dot T4SS, which translocates approximately 300 different effector proteins into the host cell (12, 33–37). Since *L. pneumophila*–host interactions are defined to a large extent by the Icm/Dot apparatus, the T4SS represents a major virulence factor of *L. pneumophila*.

The Icm/Dot T4SS and translocated effectors control every step in the infection process of *L. pneumophila* (Figure 1), i.e., the uptake (38, 39), inhibition of fusion with lysosomes (40) and acidification (41), subversion of retrograde trafficking (42), as well as interception of secretory vesicle trafficking (43), coalescence with the ER (44–46), and finally, egress from the host cell (47). To interfere with host cell processes, many *L. pneumophila* effector proteins target pivotal components of eukaryotic membrane dynamics, such as phosphoinositide (PI) lipids and small GTPases (12, 33, 37). The PI lipids PtdIns(4)*P* and PtdIns(3)*P* control the secretory and the endosomal pathway, respectively, while the small GTPases Arf1 and Rab1 are pivotal regulators of ER-Golgi secretory trafficking.

Some Icm/Dot substrates subvert PI lipids by using PtdIns(4)*P* and PtdIns(3)*P* as membrane anchors, both of which are found on LCVs (33, 48–50). Accordingly, the effectors SidC, SdcA, SidM, LidA, Lpg1101, and Lpg2603 bind PtdIns(4)*P* (51–57), and the effectors LidA, LptD, RidL, SetA, and LpnE bind PtdIns(3)*P* (42, 52, 58–60). Two *L. pneumophila* “CX_5_R” domain PI phosphatases have been identified: SidF, a PI 3-phosphatase that hydrolyzes PtdIns(3,4)*P*_2_ [and also PtdIns(3,4,5)*P*_3_] *in vitro* (61), and SidP, a PI 3-phosphatase that hydrolyzes PtdIns(3)*P* as well as PtdIns(3,5)*P*_2_*in vitro* (62). Thus, these PI phosphatases might cause PtdIns(4)*P* to be formed on and PtdIns(3)*P* to be removed from LCV membranes. LppA is another Icm/Dot substrate that hydrolyzes polyphosphorylated PIs to mainly produce PtdIns(4)*P in vitro* (49). However, LppA does not affect the LCV PI pattern in infected cells, but rather functions as a translocated hexakisphosphate inositol phosphatase (phytase), which possibly promotes intracellular replication of *L. pneumophila* by removing the intracellular micronutrient chelator phytate.

Intracellular multiplication/defective for organelle trafficking T4SS substrates modulate the activity of the small GTPases Arf1 (RalF) (63) or Rab1 (SidM) (55, 64–67) by acting as guanine nucleotide exchange factors (GEFs), GTPase-activating proteins (GAPs; LepB) (68), AMPylase/deAMPylase (SidM, SidD) (69–72), or phosphocholinase/dephosphocholinase (AnkX, Lem3) (73–77). The covalent modifications of Rab1 by AMP or phosphocholine cause a prolonged activation of the GTPase, by preventing its inhibition by the GAP LepB. Similarly, the Icm/Dot substrate LidA binds to activated Rab1 and prevents inactivation by LepB, as well as covalent modifications by SidM or AnkX (64, 78–80). Finally, the PtdIns(4)*P*-binding effector SidC and its paralog SdcA promote the monoubiquitination of Rab1, in agreement with their function as an E3 ubiquitin ligase containing a catalytic Cys–His–Asp triad (81–83). In summary, after their translocation a number of *L. pneumophila* Icm/Dot substrates employ PI lipids on the cytoplasmic side of the LCV membrane as membrane anchors. Thus, PI lipids determine at least in part the subcellular localization of the effectors, which show different biochemical activities, including subversion of small GTPases, ubiquitinylation, or binding to and modulation of vesicle trafficking machinery.

## Inhibition of Retrograde Trafficking and Autophagy

Retrograde trafficking in eukaryotes comprises the transport from early or late endosomes through the *trans*-Golgi network (TGN) to the ER (84). The cation-independent mannose 6-phosphate receptor (CI-MPR) is a well-documented substrate of the retrograde transport pathway. CI-MPR binds mannose present on hydrolases and transports the cargo destined for the endocytic pathway from the TGN to the endosomal system. After releasing the cargo in the lysosomal lumen, the receptor is recycled back through the retrograde pathway to the TGN (85). Proper recycling requires a protein complex termed the retromer, composed of a cargo recognition subcomplex (Vps26, Vps29, and Vps35) and a membrane-deforming subcomplex, which consists of a dimer of sorting nexins (SNXs) (86). The cargo recognition complex is recruited to the membrane by the activated small GTPases Rab5 and Rab7 (87).

Recent findings indicate that *L. pneumophila* communicates with the retrograde trafficking pathway. The small GTPases Rab5 and Rab7 (88–91), as well as the retromer subunits Vps26, Vps29, and Vps35 (42, 92) localize to the LCV. Interestingly, the Icm/Dot T4SS-translocated substrate RidL functions as a bacterial interactor of the eukaryotic retromer complex (42). RidL, which localizes on LCVs in *D. discoideum* cells and macrophages, binds to the Vps29 subunit of the cargo recognition subcomplex of the retromer. Subunits of the retromer cargo recognition complex localize to the LCV in an Icm/Dot-dependent but RidL-independent manner. RidL competes with SNX1 and SNX2 in its capacity for membrane binding specifically through PtdIns(3)*P*, thus possibly triggering the removal of SNXs. By altering the retrograde trafficking cascade, RidL might promote the formation of a non-lysosomal replicative vacuole and intracellular replication of *L. pneumophila* in protozoan and metazoan phagocytes (42). Earlier work already indicated that the retrograde pathway might control intracellular replication of *L. pneumophila*. The PtdIns(4,5)*P*_2_ 5-phosphatase OCRL1 and its *D. discoideum* homolog Dd5P4 are implicated in retrograde trafficking (93, 94) and localize to LCVs (60). Depletion of OCRL by RNA interference (42) or deletion of Dd5P4 (60) increased intracellular growth of *L. pneumophila*, indicating that the PI 5-phosphatase OCRL/Dd5P4 indeed restricts pathogen replication.

Autophagy is a major cell-autonomous defense mechanism used by infected cells against intracellular bacteria. The process of macroautophagy is responsible for the degradation of cytoplasmic constituents, such as bacteria or damaged organelles, which are engulfed by autophagosomes and subsequently fuse with lysosomes (95). In the course of microautophagy, the constituents are directly delivered to the lysosomes. Over 30 autophagy-related genes (Atg) have been discovered during the last years (96). An Atg protein essential for autophagy is the microtubule-associated protein light chain 3 (LC3, *alias* Atg8), which is conjugated to phosphatidylethanolamine and localized on autophagosomal membranes (97). Measurement of LC3/Atg8 during *L. pneumophila* infection revealed an Icm/Dot-dependent inhibition of autophagy by the bacteria. Intriguingly, the bacterial protein RavZ was identified as an effector required for autophagy inhibition, yet further effectors could be involved, since *L. pneumophila* lacking *ravZ* still evaded the autophagy cascade (98). RavZ is a cysteine protease, which irreversibly deconjugates phosphatidylethanolamine from LC3/Atg8, thus reducing its membrane accumulation and activity. While most autophagy factors are not necessary for *L. pneumophila* replication in *D. discoideum* (99), amoebae lacking Atg9 do not internalize the pathogen as efficient as wild-type cells, yet allow more effective intracellular growth (100). In summary, these findings suggest that *L. pneumophila* inhibits retrograde vesicle trafficking as well as autophagy to promote intracellular growth.

## Modulation of Organelle Motility and Cell Migration

The Icm/Dot T4SS is crucially involved in the formation of the LCV, and many *L. pneumophila* effectors selectively decorate the pathogen vacuole membrane. Intact LCVs can be purified by a straight-forward two-step protocol involving immuno-affinity enrichment using an antibody against the *L. pneumophila* effector SidC selectively decorating the pathogen vacuole and a secondary antibody coupled to magnetic beads, followed by a conventional density centrifugation step. Proteomics analysis of purified preparations of LCVs from infected *D. discoideum* amoebae (91) and RAW 264.7 macrophages (90) revealed the presence of 670 and 1150 host proteins, respectively, including 13 small GTPases of the Rab family, as well as the small GTPase Ran and its effector Ran binding protein 1 (RanBP1).

Ran is a member of the Ras superfamily of small GTPases and is fundamental in numerous cellular processes, such as nuclear pore translocation (101), or mitotic spindle assembly and post-mitotic nuclear envelope formation (102, 103). Ran GTPase also plays an important role in cytoplasmic processes involving non-centrosomal microtubules, e.g., endocytic receptor trafficking and retrograde signaling along microtubules in nerve axons (104). Ran can be activated by a nuclear (or in mitotic cells: chromatin bound) Ran GEF termed regulator of chromosome condensation 1 (RCC1) (105). Ran(GTP) is inactivated by the cytoplasmic Ran GTPase-activating protein 1 (RanGAP1) together with RanBP1, which harbors a Ran(GTP)-binding domain (104).

Intriguingly, *L. pneumophila* produces an Icm/Dot-translocated effector termed LegG1 that harbors eukaryotic-like RCC1 domains (106, 107). The corresponding gene is conserved among the *L. pneumophila* genomes sequenced to date, including the strains Philadelphia-1, Paris, Lens, Corby, Alcoy, and Lorraine. LegG1 (Lpg1976/PieG) is encoded in the plasticity island of effectors (Pie) gene cluster (108). The C-terminal CAAX tetrapeptide motif of LegG1 is lipidated by the host prenylation machinery (109), thus promoting the membrane localization of the effector to small vesicular structures upon ectopic expression in eukaryotic cells (108).

Recent work revealed that LegG1 is indeed an *L. pneumophila* virulence factor, which promotes intracellular bacterial replication but is dispensable for uptake (110) (Figure 1). A Δ*legG1* mutant strain is not less cytotoxic than the parental strain, but outcompeted by wild-type *L. pneumophila* upon co-infection of *A. castellanii* amoebae. Moreover, Ran, RanBP1, and LegG1 accumulate in an Icm/Dot-dependent manner on the LCV, and the bacterial effector localizes to the cytosolic face of the LCV in *L. pneumophila*-infected phagocytes. *L. pneumophila* wild-type but not the Δ*legG1* mutant strain activates Ran on LCVs and in cell lysates; yet, while LegG1 promotes the accumulation of RanBP1 on LCVs, the effector is dispensable for the recruitment of the small GTPase. Several experimental approaches indicated that in infected phagocytes *L. pneumophila* triggers the polymerization of microtubules in a LegG1-dependent manner (110) (Figure 1). “Microbial microinjection” of LegG1 by *Yersinia enterocolitica* confirmed the positive effect of LegG1 on microtubule stabilization. Here, *Y. enterocolitica* harboring a T3SS, but lacking endogenous effectors, translocated LegG1 fused to a type III secretion signal into HeLa cells. Furthermore, while LCVs harboring wild-type *L. pneumophila* vividly move along microtubules in infected *D. discoideum*, the pathogen vacuole harboring Δ*legG1* mutant bacteria is stalled (110). In summary, the discovery and characterization of the *L. pneumophila* Ran activator LegG1 revealed an unexpected role of the small GTPase Ran in the formation of pathogen vacuoles.

**Figure 2:**
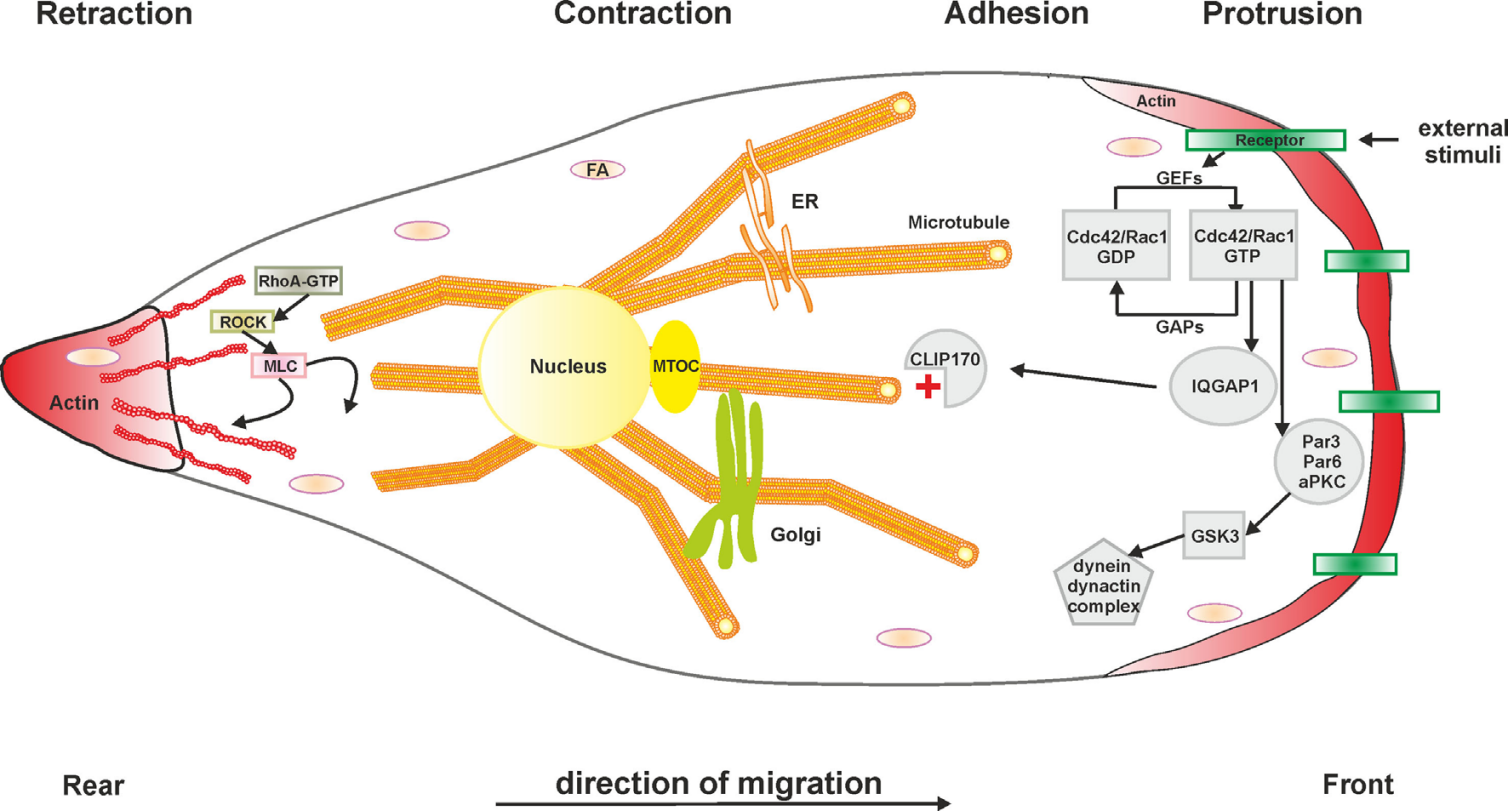
**Schematic representation of cell migration**. Generally, the cellular migration cycle can be divided into four major steps: (1) protrusion at the leading edge, (2) cell adhesion, (3) contraction to generate the required forces, and (4) retraction at the rear edge. Microtubules and the actin cytoskeleton in concert with Rho GTPases are fundamental components of each phase. Abbreviations: aPKC, atypical protein kinase C; Cdc42, cell division control protein 42; CLIP170, cytoplasmic linker protein 170; FA, focal adhesion; GAP, GTPase-activating protein; GEF, guanine nucleotide exchange factor; GSK3, glycogen synthase kinase 3; IQGAP1, IQ motif-containing GTPase-activating protein 1; MLC, myosin light chain; MTOC, microtubule organizing center; Par3/6, partitioning defective 3/6 homolog; Rac1, Ras-related C3 botulinum toxin substrate 1; RhoA, Ras homolog gene family member A; ROCK, Rho-associated protein kinase. The *L. pneumophila* effector protein LegG1 promotes cell migration by activating the small GTPase Ran and stabilizing microtubules. Other *L. pneumophila* factors modulating cell migration are not known.

Microtubule polarization and dynamics represent pivotal determinants of eukaryotic cell migration (111) (Figure 2). Given the prominent role of Ran and LegG1 on the dynamics of the microtubule cytoskeleton, we investigated the effect of *L. pneumophila* and LegG1 on host cell motility (112). Studies using *D. discoideum* amoebae or immune cells, such as RAW 264.7 macrophage-like cells or primary PMN, in different migration assays (under-agarose and Boyden chamber assays), revealed an Icm/Dot-dependent inhibition of migration. Phagocytes infected with wild-type *L. pneumophila* or *Legionella longbeachae* showed a substantially reduced migration when compared to cells lacking a functional Icm/Dot T4SS. Uptake and cytotoxicity assays demonstrated that the observed effect is not due to a defect in infection.

Based on these findings, the modulation of cell migration by LegG1 was assessed in *D. discoideum* as well as in RAW 264.7 macrophages or PMN (112) (Figure 1). Interestingly, the Δ*legG1* mutant strain hyper-inhibited the directed migration of phagocytes in the under-agarose assay, even to a larger extent than wild-type *L. pneumophila*. Overproduction of LegG1 in the Δ*legG1* mutant background re-established the migration range to an extent comparable to cells infected with a Δ*icmT* mutant strain. Single cell tracking revealed that the forward migration and the velocity of cells infected with wild-type or Δ*legG1 L. pneumophila* was impaired. Similarly, upon “microbial microinjection” by *Y. enterocolitica*, LegG1 was sufficient to stimulate migration of epithelial cells in scratch assays. Moreover, using RNA interference a role of Ran in the LegG1-dependent migration inhibition was demonstrated. Upon depletion of Ran, cells infected with the strain overproducing LegG1 were not able to migrate and to close scratch wounds over time (112). Taken together, the Ran activator LegG1 promotes cell motility by modulating microtubule dynamics and thus antagonizes Icm/Dot-dependent inhibition of cell migration. LegG1 might reverse the otherwise deleterious impact of other *L. pneumophila* effectors on the host cytoskeleton, and thereby sustain vesicle trafficking and organelle motility required for the establishment and maintenance of LCVs.

## Triggering Inflammasomes and Programed Cell Death

Maintenance of LCV integrity and regulation of programed cell death is critical for preserving the intracellular replication niche of *L. pneumophila* (113). Accordingly, impaired activation of programed cell death turned out to account for the failure of A/J mice to restrict *L. pneumophila*. The allele conferring sensitivity against *L. pneumophila* was mapped to the *NAIP5* gene (114, 115) within the *Lgn1* locus (116). The Naip5 (Birc1e)/Nlrc4 (Ipaf) inflammasome recognizes flagellin and triggers caspase-1 activation, pore formation, and pyroptosis (117–125). Naip5/Nlrc4 inflammasome activation represents a crucial mechanism of *L. pneumophila* restriction. In agreement with this notion, macrophages as well as DC restrict *L. pneumophila* replication through a cell death pathway mediated by Naip5, caspase-1, and caspase-3 (126). Yet, neither inflammasomes nor caspases are conserved in amoebae (127).

In addition to PAMPs, such as flagellin, a number of specific Icm/Dot substrates are implicated in the regulation of programed host cell death. The *L. pneumophila* PI 3-phosphatase SidF inactivates the pro-apoptotic factors BNIP3 and Bcl-rambo by an unknown mechanism, and thereby counteracts cell death induction (128). Furthermore, the effector SdhA plays a role in maintaining the LCV membrane integrity and also contributes to the prevention of cell death (129, 130). Cell death induction in absence of *sdhA* is suppressed by a secreted bacterial phospholipase A through an unknown mechanism. *L. pneumophila* lacking *sdhA* resides in the cytoplasm and triggers caspase-1 activation and IL-1β secretion, as well as macrophage pyroptosis through the DNA-sensing AIM2 inflammasome (131). The Icm/Dot substrate SdhA is also a key suppressor of the type I IFN (IFN-α/β) response to *L. pneumophila* through nucleic acid-sensing PRRs. Accordingly, RNA from *L. pneumophila* lacking *sdhA* triggers the RIG-I-dependent production of type I IFNs (132).

*Legionella pneumophila* also promotes programed cell death in an Icm/Dot-dependent manner. The phospholipase VipD destabilizes mitochondrial membranes by means of its phospholipase A activity, thus potentially contributing to the release of cytochrome *c* into the cytosol. This triggers pro-apoptotic caspase-3 activation and subsequently promotes host cell death (133). Analogously, the Icm/Dot-translocated phospholipase PlcC hydrolyzes several lipids, including phosphatidylcholine, phosphatidylglycerol, and phosphatidylinositol, which might destabilize target membranes and cause cell toxicity (134). Stimulation of apoptosis by an intracellular pathogen seems counterintuitive, but might reflect a tight spatial and temporal control of LCV maturation, followed by the release of the bacteria from the pathogen compartment and the host cell at the end of an infection cycle. Thus, the elaborate coordination of anti- and pro-apoptotic factors optimally supports intracellular bacterial proliferation.

Finally, caspases not only regulate cell death during *L. pneumophila* infection but also control vesicle trafficking pathways and thus contribute to the formation of LCVs. Dependent on the Nlrc4 inflammasome and Naip5, *L. pneumophila* activates caspase-7 downstream of caspase-1, and consequently, the pathogen is delivered to lysosomes (117, 135). In turn, upon deletion of the caspases, *L. pneumophila* forms an ER-derived replicative compartment. Similarly (but independently of caspase-1), active caspase-11 restricts the replication of *L. pneumophila* by promoting the fusion of the pathogen compartment with lysosomes (136).

## Activation of the Transcription Factor NF-κB

The transcription factor NF-κB is a master regulator of the mammalian innate immune response and controls the production of anti-apoptotic and pro-survival factors as well as inflammatory mediators (137). Thus, the activation of NF-κB (which is not conserved in amoebae) represents another, more indirect way to prevent host cell death. NF-κB is composed of five Rel family proteins: RelA, RelB, c-Rel, and the precursors p100 and p105, which are processed to their mature forms, p52 and p50. The transcription factor is maintained in its inactive form in the cytoplasm by a family of inhibitors termed IκBs (IκBa, IκBb, IκBe, and Bcl-3).

Bacterial PAMPs, including LPS or flagellin, initiate the NF-κB cascade through TLR2 or TLR5 and the adaptor MyD88. *L. pneumophila* triggers the production of pro-inflammatory cytokines, such as IL-1β, and the activation of the inflammasome platform through these receptors (138–141). The deletion of single TLRs does not dramatically alter *L. pneumophila* infection; yet, mice lacking MyD88 fail to produce cytokines, such as NK cell-derived IFN-γ, and are highly susceptible to *L. pneumophila* infection (142–144, 29). Moreover, mice lacking the IL-1 receptor are impaired for PMN recruitment and bacterial clearance (145). Therefore, IL-1R/MyD88-dependant signaling is critical for host resistance to *L. pneumophila* infection.

The NF-κB pathway can be activated not only by bacterial PAMPs via a TLR-dependent pathway but also by distinct bacterial effectors (146). The Icm/Dot T4SS and its substrates LnaB and LegK1 strongly induce NF-κB (147–149). While the molecular mechanism of the novel effector LnaB is not understood, LegK1 is a Ser/Thr kinase that phosphorylates the NF-κB inhibitor IκBα, leading to a robust NF-κB activation by triggering the release and nuclear translocation of the transcription factor (150). Since *L. pneumophila* lacking *legK1* is not impaired for intracellular replication, other effectors might also modulate the NF-κB response. Among the five *L. pneumophila* Icm/Dot substrates that show protein kinase activity *in vitro* (LegK1–LegK5), LegK2 is a virulence factor, which promotes intracellular replication and efficient recruitment of the ER to LCVs (151).

Translation inhibition by the Icm/Dot substrates Lgt1-3, SidI, and SidL also specifically decreases the production of IκB, even though the effectors are overall cytotoxic (152, 153). To shut down translation, the UDP-glucosyltransferases Lgt1–3 modify the elongation factor eEF1A (154, 155), and SidI inactivates eEF1A/eEF1Bγ by an unknown mechanism (153). Finally, *L. pneumophila* also modulates host transcription more directly. The Icm/Dot substrate RomA/LegAS4 promotes intracellular replication as a SET domain-containing histone methyltransferase that modifies (immune) gene expression (156, 157). In summary, *L. pneumophila* modulates protein production and turnover through the activation of NF-κB and by altering the epigenetic pattern, as well as through the inhibition of translation.

## Conclusion and Outlook

The ubiquitous environmental bacterium *L. pneumophila* triggers an acute and potential fatal pneumonia termed Legionnaires’ disease. The opportunistic pathogen employs the Icm/Dot T4SS and as many as 300 different effector proteins to govern interactions with phagocytes and form an intracellular replication niche, the LCV. Some Icm/Dot-translocated effector proteins interfere with (i) endocytic, secretory, or retrograde vesicle trafficking pathways, (ii) organelle or cell motility, (iii) the inflammasome and programed cell death, or (iv) the transcription factor NF-κB. Future studies will address the molecular mechanisms of action of the many Icm/Dot substrates, which remain uncharacterized to date. The further analysis of *L. pneumophila* effector proteins will likely continue to provide novel insights into the elaborate pathogen–host interactions between a highly adapted opportunistic pathogen and its phagocytic host cells.

## Conflict of Interest Statement

The authors declare that the research was conducted in the absence of any commercial or financial relationships that could be construed as a potential conflict of interest.
